# Traumatic events, PTSD, and psychiatric comorbidity in forensic patients – assessed by questionnaires and diagnostic interview

**DOI:** 10.1186/1745-0179-2-7

**Published:** 2006-04-04

**Authors:** Samia Sirag Garieballa, Maggie Schauer, Frank Neuner, Evangelia Saleptsi, Tilman Kluttig, Thomas Elbert, Klaus Hoffmann, Brigitte S Rockstroh

**Affiliations:** 1 Department of Psychology, University of Konstanz, and Centre for Psychiatry Reichenau, Germany

## Abstract

**Background:**

Relationships between posttraumatic stress disorder (PTSD), comorbid illness and experiences of traumatic stressors have been reported for large and different groups. The present study investigated this relationship specifically for patients with psychiatric disorders admitted to a forensic ward because of criminal behavior.

**Methods:**

In sixteen German and fifteen Sudanese forensic patients the prevalence of PTSD and comorbid symptoms of anxiety and depression were assessed and related to traumatic experiences, emotional distress, and stressful life events over four developmental periods.

**Results:**

In the total sample, subjects had experienced an average of five traumatic events, the first one occurring early in childhood, and 39% met criteria of current, 55% of lifetime PTSD, the diagnosis being more likely in patients with a greater number of reported traumatic experiences. Neglect and emotional abuse in childhood were associated with current PTSD diagnosis. As reported for other populations, comorbid symptoms were frequent with 60% of the sample displaying comorbid anxiety symptoms and 64% comorbid depression. PTSD and comorbidity did not differ between cultures.

**Conclusion:**

Results suggest that forensic patients experience multiple traumatic events, usually beginning early in development, so that the assessment of PTSD and comorbid anxiety and depression is recommended for the clinical evaluation. Further studies have to substantiate, whether traumatic stress during developmental stages interact with other factors leading to routes of forensic psychopathology.

## Background

Mental illness as a consequence of traumatic experiences, like posttraumatic stress disorder (PTSD), has been reported for large and varied populations [1-4]. However, evidence for forensic patients, that is individuals who committed a crime but were assigned to psychiatric treatment because of psychiatric or personality disorders, is less extensive though of particular interest. Forensic patients are often admitted because of crimes including violence, so that evidence of a relationship between earlier traumatic experience of, for instance, violence and crime, and PTSD might improve our understanding of forensic development. Traumatic experience and violence might be connected in a vicious circle, in which experience of violence might promote PTSD, and symptoms of PTSD like hyperarousal, the readiness for attack, anger outbursts, flashbacks triggered by conditions similar to those of the traumatic experience, might pose a risk factor for uncontrolled violence and criminal acts [5,6]. Indeed, the rate of traumatic experiences and PTSD has been reported to be higher in samples of delinquent subjects and prison inmates than in the general population [7-12]. Moreover, criminal offenses have been linked to negative or traumatic experiences in childhood, the trauma including the witnessing of interpersonal violence [8], and personality disorders are discussed as mediators between childhood experiences and adult delinquency [13-16].

Over the last decade, interest in the study of traumatic events and PTSD in forensic patients has increased: For instance, Stone [17] concluded from the biographies of 42 serial murderers that low socio-economic status, broken home, parental neglect, brutality, or alcoholism were common experiences in 90% of the sample. Similarly, Lewis and colleagues [18] reported a history of severe physical and/or sexual abuse during childhood in 12 murderers with dissociative identity disorder. Timmerman and Emmelkamp [19] examined the relationship between traumatic experiences, dissociation, and borderline personality disorder in 39 male forensic patients and 192 male prisoners; they found at least one traumatic event in 28% of the forensic sample, and more experiences of emotional and sexual abuse among forensic patients (>40%) than among prisoners (<29%). Similarly, Spitzer and colleagues [20] reported at least one traumatic event in 75% of 53 German forensic patients, 17% of them met the criteria for current PTSD and 56% met the criteria for a lifetime diagnosis of the same disorder. Regarding comorbid mental disorders, lifetime and current rates of anxiety disorders were found to be higher in prison inmates with than without PTSD [12]. In forensic patients, however, knowledge about comorbid psychopathology seems insufficient so far.

The present study aimed at extending evidence by assessing traumatic experiences across life together with related disorders in forensic patients. Two cultural settings were compared, a German and a Sudanese sample, in order to examine whether and how social conditions might affect symptom profiles. The prevalence of PTSD was related to (1) type and frequency of traumatic and aversive experiences across developmental periods, and (2) comorbid symptoms of anxiety and depression.

## Methods

### Subjects

Subjects were recruited from the forensic unit of the Center for Psychiatry Reichenau, Germany, and from the forensic unit of the Hospital Center for Psychiatry, Khartoum, Sudan. Both hospitals receive the majority of patients from the district they provide with psychiatric community care. Patients were included in the sample, if they (1) were between 18 and 65 years of age, (2) showed adequate mental capacity to engage in an interview and self-report questionnaires, and (3) gave informed consent confirming willingness to participate.

The sample included 16 German subjects (12 males and 4 females; mean age 39.3 ± 9.3 years, range 21–64 years) who were admitted to the forensic unit at the Center for Psychiatry Reichenau, and 15 Sudanese male subjects (mean age 32.6 ± 8.0 years, range 21–52 years) who were admitted to the forensic unit of the Hospital Center for Psychiatry Khartoum. From the German sample, 56% had completed elementary school, which held true for 40% of the Sudanese group, and 31.2% of the German group along with 27% of the Sudanese group had completed secondary school. Patients were admitted to the forensic units with one of the following DSM-IV diagnoses: (1) a personality disorder (German: 56%, Sudanese: 13%), (2) schizophrenia (German: 19%, Sudanese: 47%), and (3) major depressive disorder (German: 13%, Sudanese: 20%). Interestingly, a PTSD diagnosis was not given to any subject by the diagnosing psychiatrist upon admission to the forensic unit. About 50% of the total sample had been convicted of violent offences against other persons.

### Assessment of PTSD, traumatic experiences and comorbid symptoms

PTSD was assessed by means of section P of the semi-structured Clinical Interview for the DSM-IV (SCID). Interviews were applied by experienced psychologists and psychiatrists, who were particularly trained in administering the SCID. All interviewers had considerable experience with diagnosing patients with trauma-spectrum disorders in different countries. In addition to the questions screening for type and frequency of traumatic events and worst event, experiences across lifespan were assessed with the Traumatic Antecedents Questionnaire, TAQ [21-26]. The TAQ asks for the frequency (never, rarely, commonly) of experiences assigned to 11 domains (ranging from positive experiences like competence and safety, to negative experiences such as neglect, physical, emotional, sexual abuse, and witnessing trauma), separately assessed for four developmental periods including early childhood (0–6), middle childhood, (7–12), adolescence (13–18), and adults (19+).

Comorbid symptoms of anxiety and depression were assessed with the Hopkins Symptoms Checklist-25 (HSCL-25) [27] and the Beck Depression Inventory [28]. German versions BDI and HSCL were available. The TAQ was translated from the original English version into German and Arabic by the authors. For the Sudanese sample, the questions used started from earlier work with Sudanese samples [29]. In this earlier project, bilingual translators were trained in the concepts of PTSD and depression. Then translation and blind back translation was performed for core questions of the semi-structured PTSD interview, the BDI and the HSCL. Questions for which the outcome of the back translation was unsatisfactory were submitted to further discussions and then to the same procedure of translation and back translation. Where necessary, questions were adjusted to the local version of Arabic by SG (whose native language is Arabic and who is fluent in English). Sudanese participants were interviewed by SG in Arabic language.

### Procedure

Subjects were recruited by the therapists of the respective units. Prior to the assessment, subjects were informed by the investigators about the purpose of the study and signed an informed consent. Then, patients filled in the TAQ, HSCL-25 and BDI, while the diagnostic interview (SCID-P) was scheduled on a different day. In the German sample, the TAQ was re-administered after 3–6 months to confirm its reliability for the forensic sample.

### Data analyses

Chi square analysis served to compare the frequency of PTSD diagnoses determined from the SCID-P between the German and the Sudanese subsamples to compare comorbid anxiety, depression and emotional distress of clinical significance (HSCL-scores above 1.75 as suggested by 27) between subjects with and without PTSD. Comparisons of the German and the Sudanese subsamples were restricted to male subjects, as there were no female patients in the Sudanese sample. Differences in type and frequency of experienced events between subgroups with and without PTSD were verified by analyses of variance (ANOVA).

In the German sample, the stability of the TAQ was evaluated by re-test over a 3–6 month interval. For the total score (sum across all domains and developmental periods), for each of the 11 event domains, and each of the four developmental periods, scores were correlated between the first and the second measurement.

## Results

According to the SCID-P interview, all subjects had experienced at least one traumatic event in their lives. With a median of 8 years, the first traumatic incidence occurred early in life. The number of traumatic experiences was somewhat lower in the Sudanese (mean 4.0 ± .92, range 3–6) than in the German (5.83 ± 2.6, range 1–9) sample of male patients (t(26) = 2.5, p < .05). The number of traumatic events in the German female patients ranged from 7–11 (8.0 ± 2.0), with a dominance of sexual assault. Events such as physical abuse and assault during childhood, witnessing traumas experienced by others, serious illness or an operation, serious accident, physical attacks or weapon threats were reported by over 50% of the total sample. Physical abuse during childhood was reported significantly more often by German than by Sudanese men (83.3% vs. 26.7, chi^2 ^= 8.5, p < .01) as was unwanted sexually offending behavior (50% vs. 6.7%, chi^2 ^= 6.5, p < .05). Sudanese men reported significantly more natural disasters as a traumatic experience than Germans (chi^2 ^= 4.9, p < .05).

Of the total sample, 17 patients met the criteria of lifetime and 12 of current PTSD, indicating prevalence rates of 54.8% and 38.7%, respectively. The German and Sudanese samples did not differ with respect to PTSD prevalence or the ratio of current and lifetime PTSD. Across the German and the Sudanese subsamples, patients with PTSD (compared to patients without PTSD) diagnoses were characterized by

**Figure 1:**
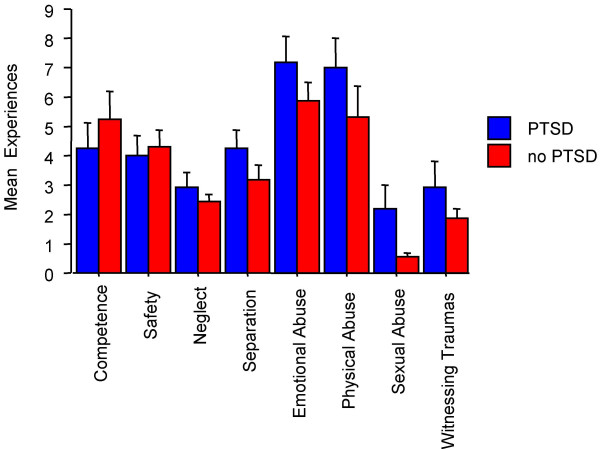
Average number of experiences separately for the 11 domains assessed by the TAQ and separately for patients with (blue) and without (red bars) current PTSD.

**Figure 2:**
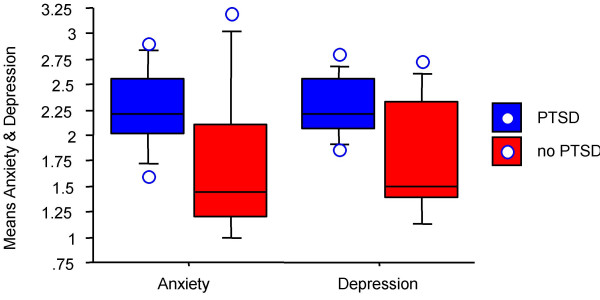
Box plot of average HSCL-25 scores of anxiety and depression in patients with (blue) and without (red) current PTSD.

a) a higher number of traumatic experiences reported in the SCID-P (6.25 ± 2.4 vs 4.6 ± 2.0; F (1,29) = 4.3, p < .05);

b) a higher frequency of sexual traumata (analyzed for male subjects only; chi^2 ^= 8.31, p < .01; of the eleven subjects who reported sexual assault, eight were currently suffering from PTSD; The mean number of experiences differed significantly between patients with and without PTSD with respect to sexual abuse (F(1,22)= 4.6, p < .05).

c) a higher frequency of negative experiences across the life span as notified in the TAQ (see Figure 1). Compared to patients without PTSD, those with PTSD had experienced significantly more neglect in early childhood (F(1,19) = 6.4, p < .05) and emotional abuse in middle childhood (F(1,19) = 4.7, p < .05);

d) higher rates of comorbid symptoms of anxiety and depression (Figure 2). More patients with current PTDS, as opposed to those without, displayed scores above the cutoff of 1.75 on the HSCL-anxiety scale (chi^2 ^= 7.81, p < .01), the HSCL-depression scale (chi^2 ^= 11.04, p < .001), and the HSCL-emotional distress scale (chi^2 ^= 11.04, p < .001). PTSD frequently occurred together with depression, as indicated by a high number of PTSD subjects with BDI score above a cutoff of 10 (chi^2 ^= 6.48, p < .05; respective differences between patients with and without lifetime PTSD were chi^2 ^= 8.76 (anxiety), 11.50 (depression), 11.50 (emotional distress), and 10.53 (BDI), all p < .01).

**Table 1:** Mean number (M ± SD in brackets) of life events according to the 11 TAQ categories, separately for four developmental periods (early childhood: 0–6 years of age; middle childhood: 7–12 years, adolescence: 13–18 years, adulthood: 19 years and older) and separately for patients with (top lines, 'yes:') and without current PTSD (lower lines: 'no'). Significant differences are marked by * (p < .05)

	0–6 yrs	7–12 yrs	13–18 yrs	(>19) yrs
Competence	yes: 0.69(.70)	1.32(1.1)	1.32(.87)	1.14(1.1)
	no: 1.29(.94)	1.42(.97)	1.50(.98)	1.46(.96)
Safety	yes: 0.87(.57)	0.94(.80)	1.24(.88)	1.06(.47)
	no: 1.26(.67)	1.17(.54)	1.22(.61)	0.94(.31)
Neglect	yes: 0.98(.45)*	1.07(.51)	1.09(.72)	1.18(.52)
	no: **0.57(.30**)	0.87(.35)	1.13(.30)	1.36(.64)
Separation	yes: 0.25(.35)	1.07(.81)	1.16(.81)	1.79(.93)
	no: 0.33(.49)	0.58(.66)	0.96(.84)	1.58(.92)
Secrets	yes: 0.56(.50)	1.35(.91)	1.41(1.0)	1.27(1.1)
	no: 0.85(.83)	1.08(.63)	1.46(.63)	1.54(.69)
Emotional abuse	yes: 0.98(.72)	1.34(.58)*	1.80(.74)	1.42(.69)
	no: 0.72(.59)	**0.88(.43**)	1.42(.63)	1.45(.51)
Physical abuse	yes: 0.50(.59)	1.09(.88)	1.51(1.14)	1.45(.88)
	no: 0.28(.45)	0.81(.93)	1.47(1.01)	1.19(.88)
Sexual abuse	yes: 0.42(1.0)	0.57(.92)	0.86(1.1)	0.43(.75)
	no: 0.00(.00)	0.21(.38)	0.33(.53)	0.04(.10)
Other trauma	yes: 0.183(.242)	0.36(.27)	0.71(.55)	1.11(.49)
	no: 0.27(.41)	0.24(.30)	0.49(.52)	1.24(.66)
Witnessing	yes: 0.56(.52)	0.79(.73)	0.94(.96)	0.82(.87)
	no: 0.26(.31)	0.51(.38)	0.67(.46)	0.58(.50)
Alcohol/drug abuse	yes: 0.19(.53)	0.50(.71)	1.04(.99)	1.18(1.1)
	no: 0.08(.28)	0.29(.54)	1.21(.99)	1.00(.98)

In the German sample, TAQ-reports of experiences across the life span were reliable, as indicated by the re-test correlation of r = .80 (p < .01) for the total score. Across developmental periods the correlation coefficients were higher for more severe events (emotional, physical abuse, other traumas, alcohol and drug abuse: r = .85–.88, p < .05), with the highest reliability found for witnessing trauma (r = .95, p < .01). In contrast, reliability of reports of sexual abuse and neglect during the early developmental periods failed to reach significance (r = .32 to .77).

Characteristics of subjects with and without PTSD did not differ between the German and the Sudanese samples, and PTSD diagnosis did not vary with the cultural setting. Only a few variables distinguished German men from Sudanese men: German subjects, for instance, reported more traumatic events than Sudanese subjects (t(23) = 2.5, p < .05), along with more safety and competence experiences during childhood and adolescence (p < .05). Physical abuse and secrets during childhood were reported more often by Sudanese than by Germans (p < .05).

## Discussion

Without exception, forensic patients in the present study reported exposure to at least one traumatic event serious enough to potentially lead to PTSD. This is in line with previous reports of higher rates of PTSD in forensic patients compared to the general population or compared to prison inmates [17-20] Also in line with previous reports, abuse (physical, sexual, emotional) was a prominent experience [30,11,12,31], which was frequently reported for childhood and early adolescence. The present findings may add to the notion of Rivera and Widom [32,15,33] that childhood victimization may favor trauma-spectrum disorders and also further later criminal behavior. However, it must be kept in mind that ours and basically all available data are only correlational and thus allow equally well alternative causal relationships. For instance, it could be that early signs of conductive disorder or delinquent behavior in a child may favor physical abuse and neglect. A viscous circle, whereby both processes would reinforce each other is also possible.

Traumatic experiences have devastating consequences: Relative to the prevalence of PTSD in the respective countries ([34] for a German sample, [35] for different Sudanese populations), and in prison inmates (<29%, [19,35,9]), the findings of 39% for current PTSD (39%) and 55% for lifetime seems alarmingly high. It has been shown (in other populations, [36,37]), that the likelihood of PTSD increases with increasing exposure to traumatic experiences (described as a "building block effect"). For forensic patients, we might assume that the multiple experiences of potentially traumatic events across the life span increase the risk for the development PTSD, potentially also for the development of a personality disorder, which is often found in forensic patients and which is often reported as comorbid diagnosis of a PTSD [26,38].

Subjects suffered substantially from anxiety, depression, and emotional distress, with comorbid symptoms being more pronounced in the present sample than in inmates of other studies, e.g. [39,25,41,12]. This result asks for particular attention for the severity of the disorder in forensic patients – with therapeutic implications, and for a clarification of the network of traumatic experiences and their consequences across the life span in larger samples and ideally prospective or longitudinal approaches.

The present results are based on retrospective self-report, limiting the power of conclusions. There has been considerable controversy about the validity of information obtained from retrospective self-reports of childhood traumata and experiences, e.g. [14,24,42-47]. Retrospective reports particularly suffer from distortion and loss of information associated with the recollection of events from a prior time period, especially those from the distant past [48,49]. On the other hand, Brewin and colleagues [50] concluded that there is little reason to link psychiatric pathology with less reliable or less valid recall of life experiences. And indeed, the test-retest reliability of the present TAQ data was high. Given that it is unlikely, that patients remembered correct assignment of events to the different developmental periods, had they just made up the data, it seems that these reflect the subjective truth.

Differences between the German and the Sudanese subsamples were small, given the common assumption of notable cultural differences in psychopathological symptoms. This suggests once more a general impact of traumatic experiences on mental health and negligible cultural modulations of the impact of negative childhood and traumatic experiences.

## Conclusion

The present results emphasize, but also suggest further investigation of the consequences of early exposure to traumatic experiences: it remains to be investigated, how personality and PTSD relate to criminal behavior, ultimately leading to a forensic status. Many mental health workers and professionals acknowledge the severe mental disorder (including comorbid depression and anxiety) in forensic patients in treatment and rehabilitation. Early detection of traumatic experiences may be among the prerequisites to prevent the development of trauma-spectrum disorders and its behavioral and clinical consequences.

## Abbreviations

DSM: Diagnostic and Statistical Manual of Mental Disorders [51]

PTSD: Posttraumatic Stress Disorder

SCID: semi-structured Clinical Interview for the DSM-IV

TAQ: Traumatic antecedent questionnaire

HSCL: Hopkins symptoms checklist

BDI: Beck depression inventory

## Declaration of competing interests

The author(s) declare that they have no competing interests.

## Authors' contributions

SG was responsible for recruitment and assessment of the Sudanese sample and participated in the data collection in the German sample, MS, FN, TE, and BR designed the study and supervised data collection and analysis, ES assisted in data collection of the German sample, TK and KH recruited patients of the German sample and had medical responsibility of the study, BR and TE supervised data analyses and wrote the article.
